# Transcatheter aortic valve-in-valve implantation in right ventricle-aorta conduit in an adult patient with Fontan circulation

**DOI:** 10.1016/j.ijcchd.2023.100476

**Published:** 2023-09-17

**Authors:** Marieke Nederend, Frank van der Kley, Madelien V. Regeer, Regina Bökenkamp, Arend de Weger, Monique R.M. Jongbloed, Anastasia D. Egorova

**Affiliations:** a CAHAL, Center for Congenital Heart Disease Amsterdam Leiden, Leiden University Medical Center, Leiden, the Netherlands; b Department of Cardiology, Leiden University Medical Center, Leiden, the Netherlands; c Department of Pediatrics, Division of Pediatric Cardiology, Leiden University Medical Center, Leiden, the Netherlands; d Department of Cardiothoracic Surgery, Leiden University Medical Center, Leiden, the Netherlands; e Department of Anatomy & Embryology, Leiden University Medical Center, Leiden, the Netherlands

**Keywords:** Fontan circulation, TAVI, Adult congenital heart disease, Valve-in-valve, Single ventricle physiology, Catheter intervention

## Abstract

Catheter interventions can offer patient tailored solutions in high-risk congenital heart disease patients. A 21-year-old male with a Fontan circulation in the setting of unbalanced atrioventricular septal defect with a hypoplastic left ventricle and an aortic homograft connecting the right ventricular outflow tract to the ascending aorta, developed failure of the heavily calcified homograft with severe regurgitation and stenosis. He underwent three sequential transcatheter aortic valve-in-valve implantations to address the homograft failure and the subsequent paravalvular regurgitation, with satisfactory result and improved hemodynamics.

## Abbreviations

ACHD: adult congenital heart disease
AV: atrioventricular
AVSD: atrioventricular septal defect
NT-proBNP: N-terminal prohormone of brain natriuretic peptide
NYHA: New-York Heart Association Function Classification
TAVI: transfemoral aortic valve implantation
RV: right ventricle

## 1 Medical history

A 21-year-old male was seen at the outpatient clinic for adults with congenital heart disease for periodic follow-up. The patient was born at 29 + 2 weeks of gestation with an unbalanced complete AVSD with a hypoplastic left ventricle and underwent pulmonary artery banding at the age of 12 days. Biventricular repair was deemed not feasible due to imbalance of the ventricles, and a Glenn procedure (anastomosis between superior vena cava and right pulmonary artery), was performed at 9 months along with surgical closure of the dysplastic pulmonary valve. At the age of 2,5 years, he underwent a valvuloplasty of the common atrioventricular (AV)-valve due to significant regurgitation and elevated right ventricular pressures. The ventricular shunt through the AVSD was restrictive, thus hindering flow from the right ventricle (RV) to the native aorta and maintaining RV pressure overload. Due to the insertion of the chordae of the AV-valve at the ventricular crest and risk of aggravating AV-valve dysfunction, septectomy could not be performed and construction of a conduit from the RV to ensure flow to the aorta was deemed necessary. The native pulmonary stump was reopened, but the dysplastic pulmonary valve could not be restored. Therefore, a 21 mm bicuspidalized aortic homograft was implanted in the pulmonary stump connecting the right ventricular outflow tract and the native ascending aorta (modified Damus–Kaye–Stansel anastomosis) to facilitate flow to the aorta and alleviate the RV pressures. The Fontan circulation was completed at the age of 4 years using an extracardiac fenestrated conduit. At the age of 5 years, the fenestration was closed using a 5mm Amplatzer (Abbott, United Stated of America) septal occluder.

## 2 Clinical presentation and investigations

The patient reported no complaints (New-York Heart Association Function Classification (NYHA) I) and had a stable exercise capacity. Transthoracic echocardiography revealed severe regurgitation and stenosis of the aortic homograft with preserved function of the native aortic valve and progressive functional regurgitation of the common AV-valve (Fig. 2A and B, Fig. 3A and B, Fig. 4A and B). Ventricular systolic function was preserved and the flow in the Fontan tunnel and Glenn was unobstructed. The patient had preserved renal function and elevated levels of N-terminal prohormone of brain natriuretic peptide (NT-proBNP) - 785.8 ng/L (upper reference limit: 161 ng/L), which had doubled in 3 years’ time. Given the potential detrimental effects of the aortic homograft dysfunction and the secondary AV-valve regurgitation on pulmonary pressures and Fontan hemodynamics, a proactive approach was followed despite the lack of overt patient reported symptoms. Additional echocardiographic investigation revealed that due to the severity of the aortic homograft dysfunction, a preferential flow from the RV through the ventricular component of the AVSD (which was no longer restrictive and had a bidirectional flow) into the native aorta now took place, which resulted in further pressure and volume overload and worsening of the functional AV valve regurgitation (Fig. 4A–B and D-E). Computed tomography angiography showed no obstruction of the Glenn or Fontan conduit and a heavily calcified homograft, measuring 18mm at the level of the annulus, Fig. 5.

**Fig. 1:**
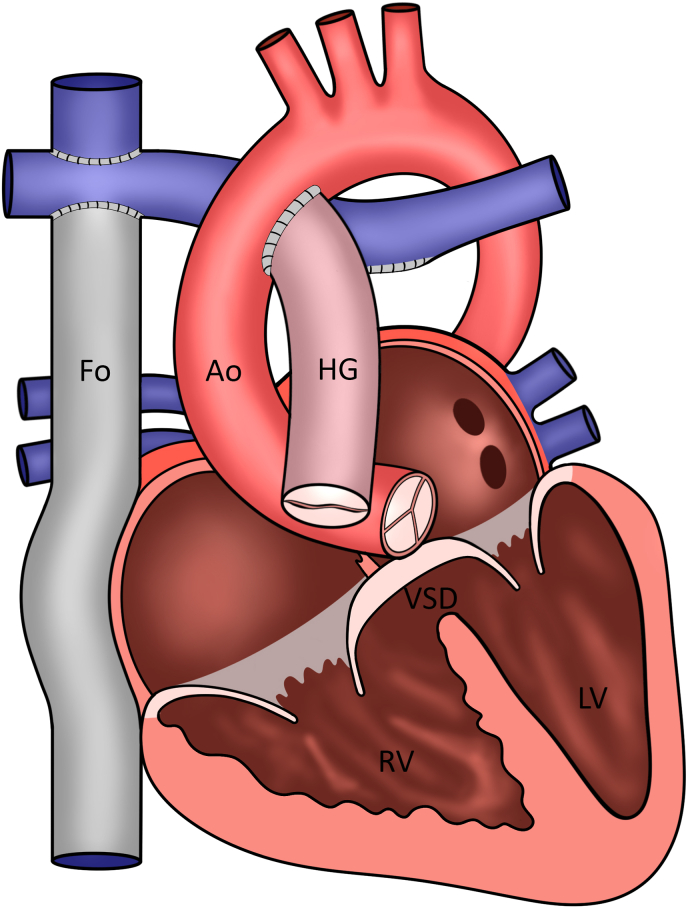
Schematic overview of patient's anatomy. Ao: aorta, Fo: Fontan, HG: homograft, LV: hypoplastic left ventricle, RV: right ventricle, VSD: ventricular component of the atrioventricular septal defect.

**Fig. 2:**
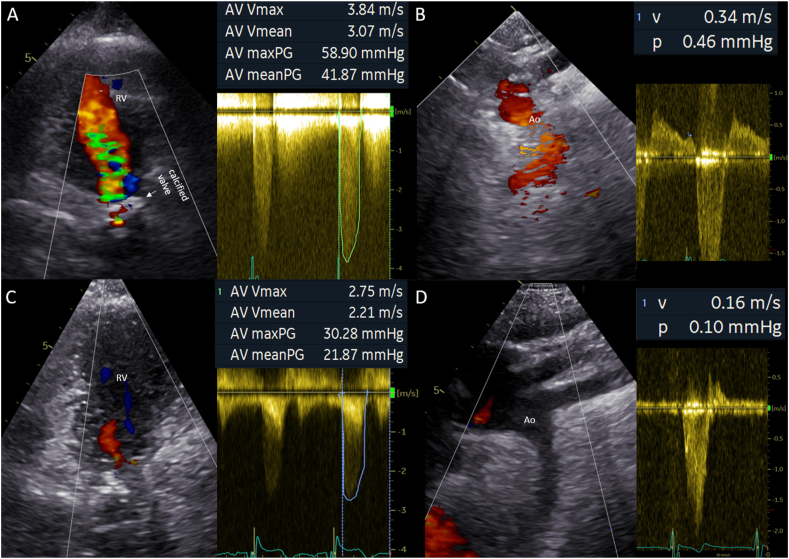
Transthoracic echocardiography imaging. Preprocedural: The modified apical two chamber view (A) diastolic frame showing the aortic homograft with severe regurgitation and severe stenosis (peak gradient 59 mmHg, mean gradient 42 mmHg). The suprasternal view (B) end-diastolic frame of the aortic arch with significant diastolic flow reversal in the descending aorta (0.34 m/s). Post-procedural, 3 months post re-intervention: The modified apical two chamber view (C) diastolic frame of the 20 mm Edwards Sapien 3 Ultra (Edwards Lifesciences, United Stated of America) bioprostheses in homograft with trace paravalvular regurgitation < grade 1 and normal gradient (peak pressure gradient 30 mmHg, mean pressure gradient 22 mmHg). The suprasternal view (D) end-diastolic frame of aortic arch with mild diastolic flow reversal (0.16 m/s). Ao: aorta, RV: right ventricle.

**Fig. 3:**
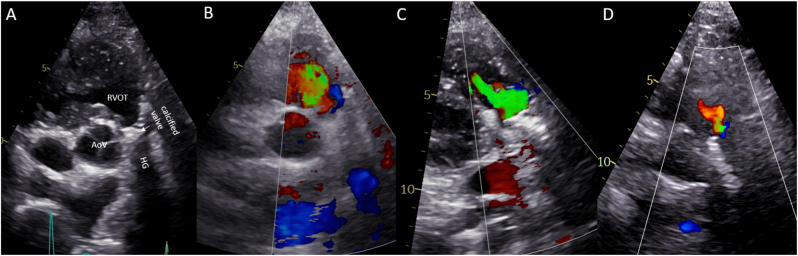
Transthoracic echocardiography imaging. Preprocedural: The parasternal short-axis view (A) illustrating the anatomy, (B) diastolic frame with massive backflow through the homograft. Post-procedural: The parasternal short-axis view (C) six weeks after first procedure showing the moderate paravalvular regurgitation, and (D) 3 months post re-intervention with trivial paravalvular regurgitation. AoV: native aortic valve, HG: homograft, RVOT: right ventricular outflow tract.

**Fig. 4:**
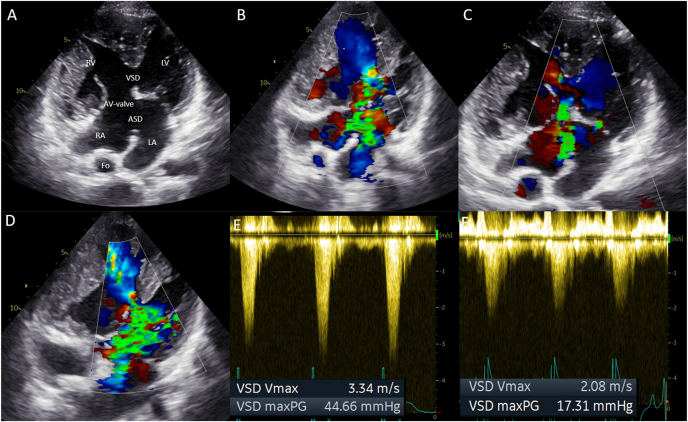
Transthoracic echocardiography imaging. Preprocedural: The four-chamber view (A) illustrating the anatomy, and (B) showing the preprocedural severe atrioventricular-valve regurgitation. Post-procedural: The four-chamber view (C) 3 months post re-intervention with the reduction of the atrioventricular-valve regurgitation to moderate. Preprocedural: The four-chamber view (D) showing the bidirectional flow over the ventricular septum defect, and (E) the flow velocity and peak pressure gradient over the ventricular septum defect (3.34 m/s, 45 mmHg). Post-procedural: The four-chamber view (F) 3 months post re-intervention showing the reduced flow velocity and peak pressure gradient over the ventricular septum defect (2.08 m/s, 17 mmHg). Note the hypertrophic right ventricle and the relatively small, hypoplastic, left ventricle. ASD: atrial shunt of the atrioventricular septal defect, AV-valve: atrioventricular-valve, Fo: Fontan conduit, LA: left atrium, LV: hypoplastic left ventricle, RA: right atrium, RV: right ventricle, VSD: ventricular component of the atrioventricular septal defect.

**Fig. 5:**
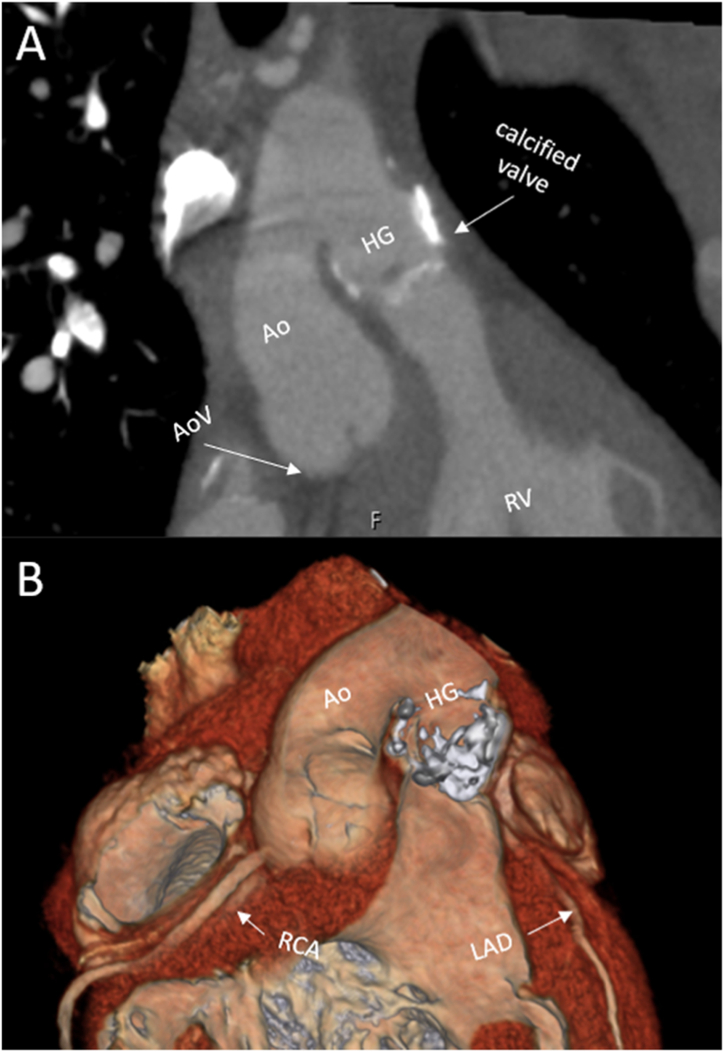
Computed tomography images illustrating (A) the severely calcified bicuspidalized homograft from the right ventricular outflow tract connected to the native ascending aorta and (B) 3D images illustrating the 3-dimensional configuration. Note the location of the coronary arteries arising from the native aorta and their respect to the homograft. Ao: aorta, AoV: native aortic valve, HG: homograft, LAD: left anterior descending artery, RCA: right coronary artery RV: right ventricle.

The patient was discussed in the congenital heart team. A weighted decision was made to pursue a percutaneous strategy given the history of four previous thoracotomies in the setting of the complex anatomy and a heavily calcified aspect of the homograft – all contributing to a high peri-operative risk and the estimated feasibility of implanting a 20mm transcatheter valve in the aorta homograft through a femoral access. The favorable anatomic characteristics excluded the risk of coronary obstruction (coronary arteries arising from the native aorta, Fig. 5) or post-procedural AV-conduction block (homograft positioned in the RV outflow tract remote from the native conduction system Fig. 1, Fig. 5).

## 3 Management

**Fig. 6:**
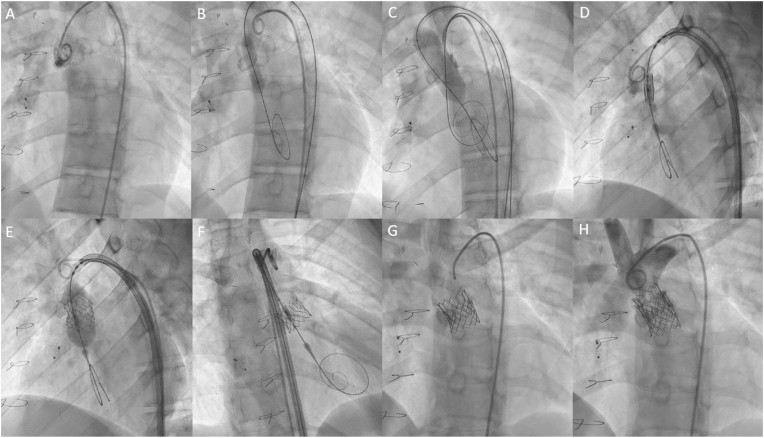
Catheterization images from transfemoral aortic valve-in-valve implantation procedures. Angiographic projection (LAO 22°) of (A) the calcified homograft, and after insertion of a Safari (Boston Scientific, United Stated of America) wire and pigtail catheter, the valve was predilated with (B) POWERFLEX® (Cordis, United Stated of America) 12mm, (C) True™ Dilatation Balloon (Becton Dickinson, United Stated of America) 18mm. Angiographic projection (LSO 43/13°) of (D) the 20 mm Edwards Sapien 3 Ultra (Edwards Lifesciences, United Stated of America) bioprosthesis positioned at the annulus level, and (E) the valve expansion with balloon. Angiographic projection (RSO 16/20°) of (F) the deployed valve. Angiographic projection (LSO 23/20°) of (G) the second 20 mm Edwards Sapien 3 Ultra (Edwards Lifesciences) bioprosthesis deployed with overlap at the annular level, and (H) the third valve deployed 20 mm Edwards Sapien 3 Ultra (Edwards Lifesciences) bioprosthesis placed superiorly reaching till the connection with the aorta.

The procedure was performed under conscious sedation through a right femoral artery access. Periprocedural echocardiography confirmed failure of the aortic homograft (severe regurgitation with diastolic backflow in the descending aorta of 0.34 m/s, mean gradient 42 mmHg, peak gradient 59 mmHg despite the preferential flow though the ventricular component of the AVSD towards the native left ventricular outflow tract and aorta, Fig. 2A and B, Fig. 4D and E). The heavily calcified homograft aortic valve was initially predilated. The 20 mm Edwards Sapien 3 Ultra (Edwards Lifesciences, United Stated of America) bioprosthesis was then positioned at the annulus level of the homograft using fluoroscopy guidance and deployed during rapid pacing over a Safari wire (Boston Scientific, United Stated of America) wire (Fig. 6A–F). Complete deployment was hampered by the heavy calcifications. After implantation, transthoracic echocardiography showed merely a trace of paravalvular regurgitation and a normal gradient over the transfemoral aortic valve (TAVI) (mean gradient 9 mmHg, peak gradient 15 mmHg). Additionally, the pressure overload of the RV was reduced, illustrated by the reduced gradient over the ventricular component of the AVSD with now solely unidirectional RV to left ventricle flow. This pressure reduction is beneficial for the end diastolic ventricular pressures, systolic and diastolic function, the Fontan pressures, and functional AV-valve regurgitation. The incomplete TAVI deployment was therefore accepted without further post dilatation attempts.

Patient was seen for follow-up 6 weeks after the procedure. He reported to be doing well, however, he had jaundice (total bilirubin 41umol/L) and signs of hemolysis were seen in the laboratory investigations with new anemia (hemoglobin 7.5mmol/L), and significantly elevated levels of reticulocytes and lactate dehydrogenase (Table 1). The hemolysis was adjudicated to the paravalvular regurgitation, which had progressed due to slight mobilization towards the ventricle (Fig. 3C). A second 20 mm Edwards Sapien 3 Ultra valve (Edwards Lifesciences) was implanted sequentially superior to the first one, ensuring some overlap. Unfortunately this did not resolve the paravalvular leakage. Consecutively, in the same procedure, a third 20mm Edwards Sapien 3 Ultra valve (Edwards Lifesciences) was required to successfully address the challenging paravalvular regurgitation (Fig. 6G and H). The patient was discharged home the following day.

**Table 1:** Laboratory values before transfemoral aortic valve-in-valve implantation (TAVI), 6 weeks post-TAVI and 3 months post re-intervention.

	Preprocedural	6 weeks follow-up	3 months follow-up
Laboratory values			
Hb (mmol/L)	9.7	7.5	8.4
MCV (fL)	92	96	83
Reticulocytes (10^9^/L)	–	195.10	69.2
Haptoglobin (g/L)	–	<0.10	0.48
Creatinine (umol/L)	94	95	85
eGFR (ml/min/1.73 m^2^)	88	86	98
LDH (U/L)	238	2105	253
ASAT (U/L)	39	136	38
ALAT (U/L)	28	39	24
AF (U/L)	102	91	104
Gamma GT (U/L)	63	54	55
Total bilirubin (umol/L)	16	41	7
NT-pro BNP (ng/L)	785.8	352.5	173.9

ALAT: alanine transaminase, ASAT: aspartase aminotransferase, eGFR: estimated glomerular filtration rate, Gamma GT: gamma glutamyltransferase, Hb: hemoglobin, LDH: Lactate dehydrogenase, MCV: mean corpuscular volume, NT-proBNP: N-terminal prohormone of brain natriuretic peptide (upper reference limit: 161 ng/L).

## 4 Follow-up

The recovery was complicated by a deep groin infection with a community acquired staphylococcus aureus, requiring surgical debridement and subsequent antibiotic treatment. At 3 months follow-up the patient is in NYHA functional class I. The NT-proBNP levels have decreased to 174 ng/L and hemolysis parameters have normalized, Table 1. Echocardiography showed good function of the TAVI, a trace of paravalvular regurgitation and moderate AV-valve regurgitation (Fig. 2C and D, Figs. 3D and 4C and F). Ventricular systolic function remained stable.

## 5 Discussion

The group of adult congenital heart disease (ACHD) patients is growing fast, with currently over 90% of children with a congenital heart defect surviving into adolescence. Patients palliated by a Fontan circulation represent the most challenging and healthcare resources reliant group due to the state of chronically elevated cardiac filling pressures and progressive cardiac dysfunction ultimately complicated by multiorgan impairment [3]. The complex anatomy, delicate hemodynamics, numerous previous surgical and catheter intervention procedures, and a notorious delay in patient perceived complaints challenge the congenital teams to create patient tailored and risk weighted management plans. In the delicate balance of the Fontan circulation, adequate timing is crucial. In the current case, the progressive rise in NT-proBNP levels and the serial increase of the functional AV-valve regurgitation was the trigger to intervene instead of to pursue a watchful waiting strategy awaiting patient symptoms or objective functional decline. In Fontan patients, AV-valve incompetence is an established marker of poor prognosis, ongoing volume overload, and deteriorating ventricular performance, rendering it an established risk factor for mortality [2].

The clinical need for catheter-based solutions to defer or postpone surgery to a later point in ACHD patients’ life is imperative. The recent European and American guidelines on ACHD patient management endorse percutaneous interventions for addressing a wide range of congenital defects, e.g.: percutaneous closure of shunt lesions, fistulae, and collaterals, balloon dilatation of the pulmonary valve or valved grafts, transcatheter pulmonary valve implantation, balloon dilatation of the aortic valve, and dilatation or stenting of narrowed great vessels and aortic coarctation [[4], [5]]. However, robust evidence on the role of percutaneous management strategies on aortic or atrioventricular valve lesions in ACHD patients is lacking and the current ESC guidelines make no recommendations in this regard[[4], [6], [7]].

Over the last decades, TAVI has emerged as a valuable treatment option for patients who may have otherwise been considered inoperable [8]. ACHD patients, and those with Fontan circulation, pose new anatomical and technical challenges in percutaneous treatment strategies, and are often excluded from clinical trials. Moharem-Elgamal et al. described the initial experience with TAVI in 13 ACHD patients, including 2 Fontan patients [9]. Results on paravalvular regurgitation, mortality and functional outcomes were promising, with no periprocedural mortality, only mild regurgitation at first follow-up 30 days after the procedure and improvement in NYHA functional class in the majority (92%) of the patients, introducing TAVI as a viable option for selected ACHD patients. Additionally, case reports of TAVI in adult single-ventricle patients for native and bioprosthetic aortic stenosis, with successful results on feasibility and outcome with improvements in NYHA class and good function of the valve have recently been reported [[1], [10]].

The current case demonstrates the potential and the challenges of TAVI in a RV outflow tract to aorta conduit in an adult patient with a Fontan circulation. The severely calcified homograft and the sharp angulation of the modified Damus–Kaye–Stansel anastomosis to the aorta posed specific technical challenges. The relief of the severe stenosis and regurgitation of the homograft resulted in a reduction of the now unidirectional (right to left ventricle) flow over the ventricular part of the AVSD. The overload of the LV and the functional AV valve regurgitation subsequently decreased improving the hemodynamic status of the patient.

## 6 Conclusions

Transcatheter aortic valve-in-valve implantation in the right ventricular outflow tract - aorta conduit in an adult patient with Fontan circulation was feasible and safe, deeming TAVI as a viable option for combined regurgitation and stenosis for a timely, less invasive, intervention to prevent potential detrimental effects of long-term volume and pressure overload of the systemic ventricle.

## 7 Learning objectives

1. To appreciate the importance of an ACHD multidisciplinary patient-oriented approach in addressing valvular interventions in Fontan patients.
2. To illustrate the potential for transcatheter valve-in-valve procedures in selected complex ACHD and univentricular physiology as an alternative in surgical high-risk patients.

## Funding and declaration of competing interest

The work was funded by the general funds of the Department of Cardiology of the 10.13039/501100005039Leiden University Medical Center, Leiden, The Netherlands. The department of Cardiology reports receiving unrestricted research and educational grants from 10.13039/100008497Boston Scientific Corporation, Medtronic, and Biotronik. The funders were not involved in study design, collection, analysis, interpretation of data, the writing of this article, or the decision to submit it for publication. ADE receives consultancy and speaker fees from 10.13039/100008497Boston Scientific Corporation and Medtronic. FK receives consultancy and speaker fees from Abbott en 10.13039/100006520Edwards Lifesciences.

## Statement of consent

All procedures performed involving the human participant were in accordance with the ethical standards of the institutional and/or national research committee and with the Helsinki declaration and its later amendments or comparable ethical standards. The patient provided written consent for publication.
